# High-resolution 3D-printed insulator-based dielectrophoresis devices for biomolecular manipulation

**DOI:** 10.1007/s00216-026-06331-6

**Published:** 2026-01-25

**Authors:** Mukul Sonker, Mohammad Towshif Rabbani, Samira Mahmud, Jorvani Cruz Villarreal, Domin Koh, Raimund Fromme, Alexandra Ros

**Affiliations:** 1 School of Molecular Sciences, Arizona State University, Tempe, AZ USA; 2 Center for Applied Structural Discovery, The Biodesign Institute, Arizona State University, Tempe, AZ USA

**Keywords:** 3D-printing, Microfluidics, Protein, DNA, COMSOL, Two-photon polymerization

## Abstract

The advancement of microfluidics has enabled a wide range of biochemical and biological applications, such as high-throughput drug testing or point-of-care diagnostics, and has also enabled dielectrophoretic applications. Dielectrophoresis (DEP) is based on the movement of polarizable particles in a non-uniform electric field. Implementing insulator-based dielectrophoresis (iDEP) in microfluidic systems has provided a new dimension for the precise manipulation of biomolecules. However, iDEP has been hampered due to the often cumbersome and expensive microfabrication methods that are required, especially for sub-µm analytes, including biomolecules, since extremely large electric field gradients are needed to achieve successful iDEP manipulation. In recent years, 3D printing has drawn attention in microfluidics, alleviating several issues with cleanroom-based fabrication methods. Among the 3D printing repertoire, two-photon polymerization (2PP) is a novel 3D printing technique that offers unique capabilities with unprecedented resolution compared to standard stereolithography. Here, we report the first iDEP-based manipulation of biomolecules, namely, λ-DNA and Phycocyanin, within a completely 3D-printed microfluidic device realized with 2PP printing. iDEP microfluidic devices with different post geometries were 3D-printed and developed with a gap resolution down to 2 µm using the IP-S photoresist. Furthermore, sub-micrometer spatial resolution was achieved down to 800 nm using the IP-Dip photoresist. Additionally, a numerical model was developed to determine the electric field gradients, DEP trapping force, and infer the associated polarizability and DEP characteristics of the analytes. This 3D printing technology may offer impactful potential for rapid prototyping of novel iDEP microdevices and the opportunity to explore iDEP for various biomolecular applications in the future.

**Graphical abstract:**

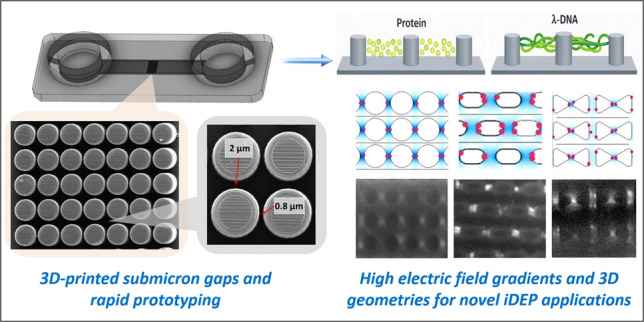

**Supplementary Information:**

The online version contains supplementary material available at 10.1007/s00216-026-06331-6.

## Introduction

Dielectrophoresis (DEP) has gained vast interest due to its non-destructive nature in precisely manipulating biomolecules and nanoparticles. DEP describes the phenomenon of a force (${{\boldsymbol{F}}}_{\mathrm{DEP}}$) experienced by a polarizable particle when it is subjected to a non-uniform electric field, and it depends on the size and dielectric properties of the particles and the suspending medium [1–4]. When a particle migrates to the higher electric field region, positive DEP (pDEP) occurs, whereas particles experiencing negative DEP (nDEP) migrate toward the lower electric field region. DEP has been successfully demonstrated for capturing and manipulating micro-to-nano particles [5–10], nucleic acids [11–13], proteins [14, 15], organelles [16, 17], cells [18–21], among other biological analytes.

Based on the mode of operation, DEP is categorized into electrode-based DEP (eDEP), contactless DEP (cDEP), and insulator-based DEP (iDEP) [3, 4, 22]. eDEP is broadly used in microfluidic devices due to several advantages, including generating high field gradients with low applied potentials. The DEP force scales with the electric field gradient ($\nabla {{\boldsymbol{E}}}^{2}$) and the volume of the particles. Thus, to manipulate the smaller-sized particles, i.e., nanoparticles, proteins, or viruses, a higher $\nabla {{\boldsymbol{E}}}^{2}$ is often required. However, eDEP devices often require demanding, multi-step fabrication workflows that rely on advanced lithographic processes and metal deposition techniques. Furthermore, the direct contact of the analyte with electrodes may result in unwanted chemical reactions and heat generation, which gave rise to cDEP applications where electrodes are not in direct contact with the analyte and are separated by an insulating barrier layer [22, 23]. iDEP offers an alternative, where insulating geometries of constrictions are introduced in a microfluidic device to generate the inhomogeneous electric field gradients. There are several advantages of iDEP over eDEP, as electrode fouling or reactions at electrodes are avoided, which also reduces bubble formation within the devices. Additionally, iDEP devices can be realized with simple fabrication methods using well-established photolithography and soft-lithography techniques [24–26]. However, achieving similarly high electric fields as in eDEP devices requires higher applied electric potentials in iDEP and cDEP devices.

To estimate required electric field gradients for successful manipulation of biological particles such as cells, viruses, and organelles, a theoretical framework to describe the polarizability mechanism has been reported previously [27–29]. For example, DEP responses for cells can be described with a shell model, which assigns different permittivities in each layer of the shell structures [3, 30]. However, theoretical models for biomolecules, such as DNA and proteins, are less developed and are just emerging, especially for proteins [2, 31–34]. Previous studies have demonstrated that extremely high electric field gradients are required to manipulate proteins and DNA using iDEP [2, 24, 32, 35, 36]. Thus, the DEP manipulation of nm-scale analytes experimentally has posed many challenges using iDEP microfluidic devices. One way to generate high electric field gradients is by applying higher electrical potentials, which can, however, induce interfering Joule heating, bubble formation, and other pronounce electrokinetic effects such as electroosmosis, electrophoresis, or concentration polarization electroosmosis (CPEO) [6, 37, 38]. In general, strategies to increase electric field gradients are based on minimizing gaps or constrictions employed in iDEP devices down to sub-µm dimensions. This process relies on nanofabrication methods such as electron beam lithography or focused ion beam milling [39–43]. However, these techniques are technically demanding and require highly trained personnel as well as specialized instrumentation within cleanroom facilities.

In recent years, additive manufacturing or three-dimensional printing (3D printing) has gained immense attention in microfluidics for its rapid prototyping capabilities and continuously improving printing speed and resolution [44, 45]. Due to advances in the field, 3D printing has been widely adopted to improve and upgrade conventional microfluidic devices, e.g., for immuno-affinity and solid-phase extraction applications [46–51], and to enable new applications in several other areas of analytical and biological chemistry [52–56]. 3D printing, when applied to the fabrication of microfluidic devices, offers several advantages over conventional photolithography [44, 57]. 3D printing alleviates several issues with cleanroom-based microfabrication and offers rapid prototyping with truly three-dimensional geometries that are not currently feasible with photolithography. This can be contrasted with soft lithography approaches using polydimethylsiloxane (PDMS), one of the most commonly used fabrication materials for iDEP devices [3, 16, 58]. Current photolithographic approaches for producing master molds for soft lithography are limited to 2D mask designs. Moreover, generating nanoscale features in PDMS demands nanofabrication equipment and a series of labor-intensive processing steps [39].

Recently, a novel 3D printing approach named two-photon polymerization (2PP) gained attention due to its unparalleled high-resolution printing capability [59]. 2PP employs a non-linear optical process where a photosensitive material absorbs two photons simultaneously to initiate a polymerization reaction in an extremely small voxel. This is opposed to stereolithography, where typically the entire resin volume in the light path is polymerized. Due to the high spatial resolution enabled by the 2PP technique, nm-gap constrictions, and the posts can be successfully integrated within such 3D-printed devices to exhibit the high electric field gradient required for the manipulation of nm-scale analytes. Here, to the best of our knowledge, we present the first report using a high-resolution, entirely 3D printed iDEP (3D-iDEP) microfluidic device manufactured by a 2PP polymerization process to dielectrophoretically manipulate biomolecules in the low-frequency regime. Furthermore, the DEP characteristics of λ-DNA and a model protein (Phycocyanin) were also studied using the 3D-iDEP devices. The experimental observations are supplemented with a numerical model, which demonstrates good agreement with previously reported polarizability values for DNA and allows for estimating the same for the protein. Such a 3D-printed iDEP device can be rapidly adapted based on analyte-of-interest, including nm-scale proteins and viruses, and can be further used to study truly three-dimensional insulating geometries for novel DEP-based separations in the future.

## Materials and methods

### Materials and chemicals

Genomic Lambda phage dsDNA (λ-DNA, 48.5 kbp) and microscope glass slides (Cover glass, No.1 Thickness, 35 × 50 mm and 24 × 40 mm) were purchased from Thermo Fisher Scientific (Waltham, MA, USA). BOBO-3 intercalating dye for fluorescent labeling of λ-DNA was obtained from Life Technologies (Carlsbad, CA, USA). SYLGARD® 184 silicone elastomer kit for polydimethylsiloxane (PDMS) was obtained from Dow Corning Corporation (Midland, MI, USA). 4-(2-Hydroxyethyl)piperazine-1-ethanesulfonic acid (HEPES), poly(ethylene glycol)-block-poly(propylene glycol)-block-poly(ethylene glycol) (Pluronic® F108), sodium hydroxide (NaOH), Dimethyl sulfoxide (DMSO), 3-[(3-Cholamidopropyl)dimethylammonio]−1-propanesulfonate hydrate (CHAPS), and sucrose were purchased from Sigma-Aldrich (St. Louis, MO, USA). Phycocyanin [60] isolated from the thermophilic cyanobacterium *Thermosynechococcus elongatus* was generously provided by Prof. Raimund Fromme (Arizona State University). Deionized (DI) water was obtained from an Elga water purification system (Woodridge, USA). Platinum wire was purchased from Alfa Aesar (Ward Hill, MA, USA). IP-Dip and IP-S photoresins were obtained from Nanoscribe GmbH (Karlsruhe, Germany).

### 3D-printed device fabrication

The device layout and channel structure were designed in Autodesk Fusion software (Autodesk, San Rafael, CA, USA) and imported into the DeScribe software of the Nanoscribe GT++ instrument (Nanoscribe GmbH, Karlsruhe, Germany). The Meso Scale printing protocol was used to print sub-5 µm resolution iDEP device structures with IP-S photoresist (Nanoscribe GmbH, Germany) using a 25 × objective in a Dip-in-liquid lithography (DiLL) configuration [61]. A small drop of IP-S was deposited on an indium tin oxide-coated boroaluminosilicate glass slide, and the designed 3D structure was printed using 2-PP polymerization of IP-S.

The device design, channel width, and post-gap dimensions were adapted as the study progressed for improved printing and development, and are shown in Figure S1 in the Electronic Supplementary Material. The initial device design (V1) consisted of an array with circular post arrays; the final device length was ~ 1.25 mm with a reservoir height and diameter of 220 µm and 450 µm, respectively. This initial design was chosen to minimize the printing time. Since this device was too small to operate experimentally, the device dimensions were further increased in the next iteration (V2). The final length of V2 was ⁓4.5 mm; the reservoir diameter was 900 µm with a height of 600 µm. The post-array channel length was 2.6 mm with a height of 50 µm. The vertical post-gaps (V-gaps) were 5 µm, whereas the horizontal post-gaps (H-gaps) varied from 5 down to 2 µm in different print iterations. Finally, a constriction was added to the channel in device V3, and the channel width was increased to facilitate ease of development and increase the achievable maximum electric field gradient, ($\nabla {{\boldsymbol{E}}}_{max}^{2}$). The sub-µm resolution of ~ 800 nm was achieved using an IP-Dip photoresist (Nanoscribe GmbH, Germany) [61, 62] with a 63× objective (Small Feature Size configuration). After printing, the devices were developed by 5 min sonication followed by 2–3 h on a shaker in SU-8 developer (Microchem, USA). The higher resolution devices required longer development time (3–3.5 h), and then, the SU-8 developer was vacuumed through the device channel to develop the abovementioned post-gaps. The developed devices were rinsed with isopropyl alcohol until complete development was observed by visual inspection with a stereomicroscope. The devices were flood-exposed for 30 min with UV radiation using a Thor Labs UV curing system to improve device robustness, and were further photobleached for 1 h using the fluorescence imaging setup to decrease the background fluorescence of the 3D printed devices before the respective imaging experiments.

A thin PDMS slab was prepared by mixing PDMS elastomer with curing agent at a 10:1 ratio (w/w), poured on a Petri dish, and degassed for 30 min. The PDMS was cured in an oven for 4 h at ~ 80 °C, and the cured cast was subsequently peeled off the Petri dish and cut into small pieces. The PDMS slab and No. 1 thickness glass slides were treated with oxygen plasma in a plasma cleaner oven (PDC-001, Harrick Plasma Cleaner, USA) at high RF (18 W) for 2 min. The PDMS slab was irreversibly bonded with the glass slide by bringing them into contact. Finally, the developed 3D-printed device was glued using epoxy glue to the PDMS slab (as shown in Fig. 3c). After assembly, the device was filled with buffer solution (1 mM F108, 10 mM HEPES, pH adjusted to 7.4 by 1 M NaOH) by capillary action. Finally, Pt electrodes were inserted in the inlet and outlets. PDMS devices for comparative studies were fabricated as described previously using an appropriate photomask [6, 16, 17]. The post array design is outlined in the “Results and discussion” section.

### Experimental setup, imaging, and data analysis

All experiments were performed using the final iteration of the 3D-iDEP devices (V2 and V3), schematically shown in Figs. 3b and S1 in the Electronic Supplementary Material. The printed microdevices were secured on the stage, and fluorescence images were acquired with an inverted microscope (IX71, Olympus, Center Valley, PA, USA) equipped with a 100 W mercury burner (U-RFL-T, Olympus, Center Valley, PA, 54 USA). The images were viewed with 20 × objectives. For the λ-DNA sample, fluorescence was collected with a filter set containing a 607/36 nm excitation filter and a 670/30 nm emission filter from Semrock (Henrietta, NY, USA). A filter set containing a 470/40 nm excitation filter and 525/50 nm emission filters (Semrock, USA) was used to image the Phycocyanin samples. Images were captured using a monochromatic QuantEM 512SC CCD camera (Photometrics, Tucson, AZ, USA) and Micro-Manager software (version 1.4.9, Vale Lab, UCSF, CA, USA). Exposure time was set to either 10 ms or 50 ms for these experiments. Pt electrodes were inserted into the inlet and outlet reservoirs and connected to an AC power supply using a high voltage amplifier (AMT-3B20, Matsusada Precision Inc.) via micro clamps (Labsmith, Livermore, CA, USA). The AC signal was produced using a USB 6343 DAQ device (USB X series, National Instruments, TX, USA) and programmed by LabVIEW 2014, version 14.0. Recorded videos and images were processed by Fiji software (version 1.53, NIH) [63]. A total of 20 to 50 trapping (pDEP) and non-trapping (nDEP) regions of interest representing high and low electric field areas were identified in Fiji for each post geometry, and the average fluorescence intensity was quantified for every region. The trapping strength was then calculated as the ratio of the mean fluorescence intensity in the trapping regions to that in the non-trapping regions for the corresponding images. Scanning electron microscopy (SEM) was performed at the Eyring Materials Center, Arizona State University, using an Auriga FIB/SEM system (Zeiss, Germany). To enable high-quality imaging, the devices were fabricated with an open architecture in the region of interest and subsequently sputter-coated with a 10–15 nm layer of gold–palladium to reduce charging and heating effects during imaging. SEM observations were conducted at an accelerating voltage of 10–20 kV, using secondary electrons for detection.

### Sample preparation

λ-DNA was diluted to 2.1 ng/µL in 5 mM phosphate buffer at pH 7.6, corresponding to the final DNA concentration of 600 pM. The λ-DNA analytes were labeled with BOBO-3 intercalating dye at a 1:10 molar ratio of dye molecules to DNA base pairs. The recovered labeled DNA was added to a 100 µL buffer. The final buffer contained 10 mM HEPES at pH 7.4, 1 mM Pluronic F108 block copolymer, 100 mM sucrose, and 0.2% v/v β-mercaptoethanol. The concentration of Phycocyanin (M_w_ 240 kDa) [60] stock solution was measured using the Nanodrop spectrophotometer (ThermoFisher Scientific, USA), Optical Density at 620 nm, and diluted to the desired final concentration using 10 mM HEPES buffer pH 7.4 containing 6.4 mM CHAPS.

### Numerical modeling

Numerical modeling was performed with COMSOL Multiphysics 6.3 to predict the trapping regions for the pDEP and nDEP cases. A section of the printed microfluidic device matching the post array geometry was drawn using AutoCAD software and was imported into COMSOL. The material properties were chosen according to pre-defined parameters for water as a medium, and a medium conductivity of 0.03 S/m. Electric potentials, *V*, were applied to assess the electric field gradient in the various design geometries, in some cases matching the electric field in the experiment, scaled to the channel section at the extremities of the geometries modeled. Details for each case are outlined in the results section. The Electric Current module was used to compute the electric field, ***E***, and ∇***E***^2^ within the section. The post walls and side walls were selected as insulators. *V* was applied to the inlet boundary, and the outlet was grounded.

Particle Tracing for the Fluid Flow module was used with a time-dependent solver to trace the trajectories and trapping behavior of the analytes, as described previously [64]. In this module, the Brownian force (${{\boldsymbol{F}}}_{b}$), drag force (${{\boldsymbol{F}}}_{D}$), and DEP force (${{\boldsymbol{F}}}_{\mathrm{DEP}}$) accounted for the total force (${{\boldsymbol{F}}}_{t}$) acting on the analytes. The electrophoretic force and electroosmotic flow were neglected in this model as they are negligible in the low-frequency regime. With the time-dependent solver, the particle trajectories were computed with the following equation [65–67]:

1 $$\frac{d({m}_{p}{\boldsymbol{v}})}{dt}={{\boldsymbol{F}}}_{t}$$

where ${m}_{p}$ and ${\boldsymbol{v}}$ are the mass and velocity of the analyte, respectively.

The Brownian force is defined as [68–72]:

2 $${{\boldsymbol{F}}}_{b}=\upzeta \sqrt{\frac{12\pi {k}_{B}\mu T{r}_{p}}{\Delta t}}$$

where ${r}_{p}$ is the radius of the analyte, $\mu$ is the viscosity, $T$ is the temperature, ${k}_{B}$ is the Boltzmann constant, $\upzeta$ is a dimensionless normally distributed random number, and $\Delta t$ is the time interval. In this model, the drag force was computed with the following equations:

3 $${{\boldsymbol{F}}}_{D}= \frac{1}{\beta }{m}_{p}({\boldsymbol{u}}-{\boldsymbol{v}})$$

4 $$\beta = \frac{{\rho }_{p}{{d}_{p}}^{2}}{18\mu }$$

where ${m}_{p}$, ${\rho }_{p}$, and ${d}_{p}$ are the mass, density, and diameter of the particle, and $\beta$ is the velocity response time. ${\boldsymbol{u}}$ and ${\boldsymbol{v}}$ refer to the fluid velocity and the particle velocity, respectively. ${{\boldsymbol{F}}}_{\mathrm{DEP}}$ was coupled using a user-defined force function accounting for the polarizability of the analyte as:

5 $${{\boldsymbol{F}}}_{\mathrm{DEP} }= \frac{1}{2}\alpha \nabla {{\boldsymbol{E}}}^{2}$$

where $\alpha$ is the polarizability of the biomolecule and ${\boldsymbol{E}}$ is the electric field [28, 29].

For λ-DNA and Phycocyanin, ${{\boldsymbol{F}}}_{\mathrm{DEP}}$ was estimated by COMSOL using Eq. 5. Using Dynamic Light Scattering (Zetasizer Ultra, Malvern Panalytical Ltd, UK), the radius of the λ-DNA and Phycocyanin was approximated as 720 nm and 3.5 nm, respectively, based on the hydrodynamic diameter measured (data not shown). An $\alpha={3.3*10}^{-29}{\mathrm{Fm}}^{2}$ was used for λ-DNA in the numerical model, as reported previously, to account for ${{\boldsymbol{F}}}_{\mathrm{DEP}}$ [29].

The $\alpha$ of Phycocyanin was unknown and estimated using the numerical model as described below. For this purpose, the minimum potentials required to trap the protein with DEP were determined experimentally. Using the applied electric field, the maximum electric field gradient $(\nabla {{\boldsymbol{E}}}_{\mathrm{max}}^{2}$) was calculated using the numerical model. Next, the $\alpha$ value was varied until trapping was observed in the numerical model. Finally, DEP trapping was obtained through the particle tracing study for a value of ${\alpha }_{\mathrm{phycocyanin}}= {3.5*10}^{-31}{\mathrm{Fm}}^{2}$. Parameters used in the numerical model can also be found in Table S1 in the Electronic Supplementary Material.

## Results and discussion

### Electric field gradient as a function of post-gap

**Fig. 1:**
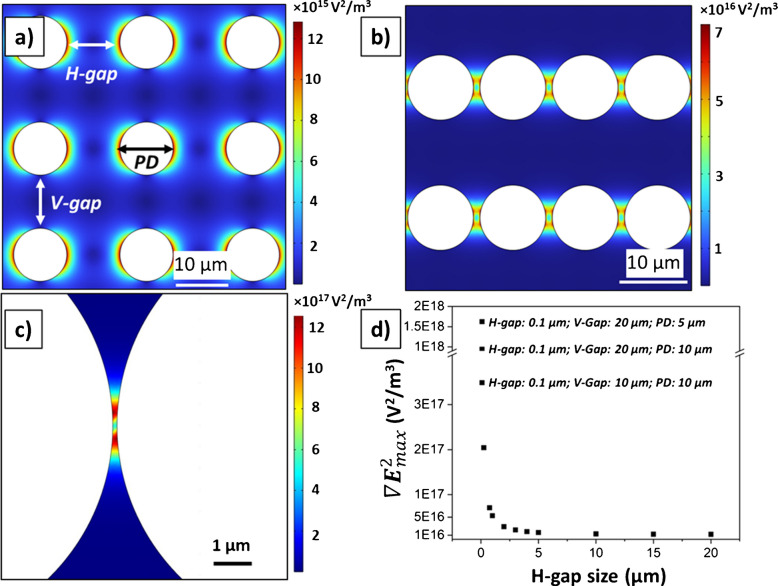
Variation in $\nabla {{\boldsymbol{E}}}_{\mathrm{max}}^{2}$ with different horizontal post gaps in a 100 × 80 µm rectangular channel. The H-gaps were varied, and the V-gap was kept at 10 or 20 µm. $\nabla {{\boldsymbol{E}}}_{\mathrm{max}}^{2}$ was computed at an applied electric field of 1000 V/cm. **a** When the H-gap and V-gap between the two circular posts were 10 µm, a $\nabla {{\boldsymbol{E}}}_{\mathrm{max}}^{2}$ of 1.28*10^16^ V^2^/m^3^ was observed, **b** the horizontal gap between the two circular posts was reduced to 1 µm (V-gap-10 µm), resulting in $\nabla {{\boldsymbol{E}}}_{\mathrm{max}}^{2}$ of 5.34*10^16^ V^2^/m^3^, **c** the horizontal gap between the two circular posts was further reduced to 100 nm, resulting in $\nabla {{\boldsymbol{E}}}_{\mathrm{max}}^{2}$ of 3.48*10^17^ V^2^/m^3^, and **d **$\nabla {{\boldsymbol{E}}}_{\mathrm{max}}^{2}$ values were predicted for various H-gaps ranging from 20 µm to 100 nm. For the case of the 100 nm H-gap, an additional V-gap of 20 µm was also studied, showing an increase in $\nabla {{\boldsymbol{E}}}_{\mathrm{max}}^{2}$ with increasing V-gap. The highest $\nabla {{\boldsymbol{E}}}_{\mathrm{max}}^{2}$ of 1.62*10^18^ V^2^/m^3^ was achieved for 5 µm post diameter (PD) with a H-gap of 0.1 µm and V-gap of 20 µm, showing > 125-fold improvement in $\nabla {{\boldsymbol{E}}}_{\mathrm{max}}^{2}$ compared to the array shown in **a**

In iDEP devices, electric field gradients can be enhanced by decreasing the gap size between constrictions or posts [39, 73]. It has been evidenced through experiments and in theory that nm-sized analyte trapping, including biomolecules as small as proteins or DNA, requires $\nabla {{\boldsymbol{E}}}^{2}$ in the range of 10^12^ to 10^21^ V^2^/m^3^ [3, 4]. Here, we explore high-resolution 3D printing to alleviate fabrication hurdles with photo- and softlithography techniques, eventually generating sub-micrometer constrictions within iDEP devices. To study the expected improvement in $\nabla {{\boldsymbol{E}}}_{\mathrm{max}}^{2}$, we first carried out a numerical modeling study in post-array devices, which were later employed for biomolecule iDEP trapping experiments. The post arrays are defined through the insulating post diameter (PD), the horizontal gap (H-gap), and the vertical gap (V-gap) as depicted in Fig. 1a. In the 2-dimensional numerical study, H-gaps ranged from 20 µm down to 100 nm in a 140 µm (L) × 80 µm (W) rectangular channel, whereas the V-gap was changed in the range from 20 to 10 µm for an applied electric field corresponding to 1000 V/cm. The highest electric field gradients and the maximum electric field obtained from these numerical models are listed in Table S2 in the Electronic Supplementary Material.

Figure 1a-c shows three examples where $\nabla {{\boldsymbol{E}}}_{\mathrm{max}}^{2}$ increased on decreasing the H-gap while the V-gap remained at 10 µm. Figure 1a**,** where both V-gap and H-gap were 10 µm, exhibits a $\nabla {{\boldsymbol{E}}}_{\mathrm{max}}^{2}$ of 1.28*10^16^ V^2^/m^3^. The $\nabla {{\boldsymbol{E}}}_{\mathrm{max}}^{2}$ increased to 5.34*10^16^ V^2^/m^3^ when the H-gap was decreased from 10 to 1 µm while keeping the V-gap at 10 µm, as shown in Fig. 1b. Figure 1c represents the case where the H-gap is further reduced to 100 nm, showing a significant increase in $\nabla {{\boldsymbol{E}}}_{\mathrm{max}}^{2}$ to 3.48*10^17^ V^2^/m^3^, over an order of magnitude higher than Fig. 1a. Figure 1d summarizes the increase in $\nabla {{\boldsymbol{E}}}_{\mathrm{max}}^{2}$ values as a function of various H-gap sizes ranging from 20 µm to 100 nm with a 10 µm V-gap. The $\nabla {{\boldsymbol{E}}}_{\mathrm{max}}^{2}$ further improved to 9.42*10^17^ V^2^/m^3^, showing over 70-fold increase, when the V-gap was increased to 20 µm, with a 100 nm H-gap, demonstrating that the vertical array spacing can also affect $\nabla {{\boldsymbol{E}}}_{\mathrm{max}}^{2}$ significantly. Moreover, we assessed the effects of the array post diameter on the $\nabla {{\boldsymbol{E}}}_{\mathrm{max}}^{2}$, demonstrating that when the post diameter was reduced from 10 to 5 µm, $\nabla {{\boldsymbol{E}}}_{\mathrm{max}}^{2}$ of 1.62*10^18^ V^2^/m^3^ was achieved. This amounts to an increase of over 125-fold compared to the initial case in Fig. 1a. The observed increase in $\nabla {{\boldsymbol{E}}}^{2}$ with decrease in post diameter arises from the increased curvature as a result of the smaller radius of the posts. Insulated regions of high curvature (smaller diameter posts) better converge electric field lines, producing stronger local field gradients. Combined with optimized H-gap and V-gap spacing, this geometric effect leads to a significant enhancement of $\nabla {{\boldsymbol{E}}}^{2}$ observed in our simulations, as smaller posts focus the electric field more efficiently and generate steeper spatial variations.

For the geometries discussed in Fig. 1, we also assessed the electric field maximum (***E***_max_). For the smallest H-gaps of 100 nm, ***E***_max_ ≥ 1*10^6^ V/m was achieved, which is well within the range of biomolecular trapping experimentally reported by others [15, 32, 74]. However, we note that more complicated constriction geometries or micrometer to nanometer-sized electrodes fabricated within microfluidic devices were employed to achieve similar electric field magnitudes [15, 32, 74]. Therefore, 2PP 3D printing offers a way to bypass complex and human error-prone microfabrication processes while producing geometries of comparable nano-scale. While the manufacturer, Nanoscribe, reports a single positive-feature resolution of less than 200 nm [75], achieving this in practice is challenging, particularly for negative features such as the inter-post gaps required for iDEP applications. Only a few studies, including our own, have successfully demonstrated sub-micrometer resolution for such negative structures [64, 76–78]. This resolution can be further enhanced to below 100 nm (for positive features) through post-processing techniques such as isotropic plasma etching or pyrolysis of printed features [79], which could also be applied in this context to create post gaps. We anticipate that ongoing improvements in photoresin chemistry, femtosecond lasers, scanning systems, and innovative post-processing methods will continue to push the achievable resolution even lower. Thus, based on the numerical assessment, we conclude that sufficiently high electric fields and gradients can be obtained within 3D-printed post-arrays providing a route to DEP-based trapping of nanoscale analytes, including proteins and viruses, while eliminating the need for traditional microfabrication workflows such as photolithography, chemical etching, metal deposition, soft lithography, or focused ion beam patterning described in earlier studies [42, 43, 58].

### iDEP trapping assessed by numerical modeling

**Fig. 2:**
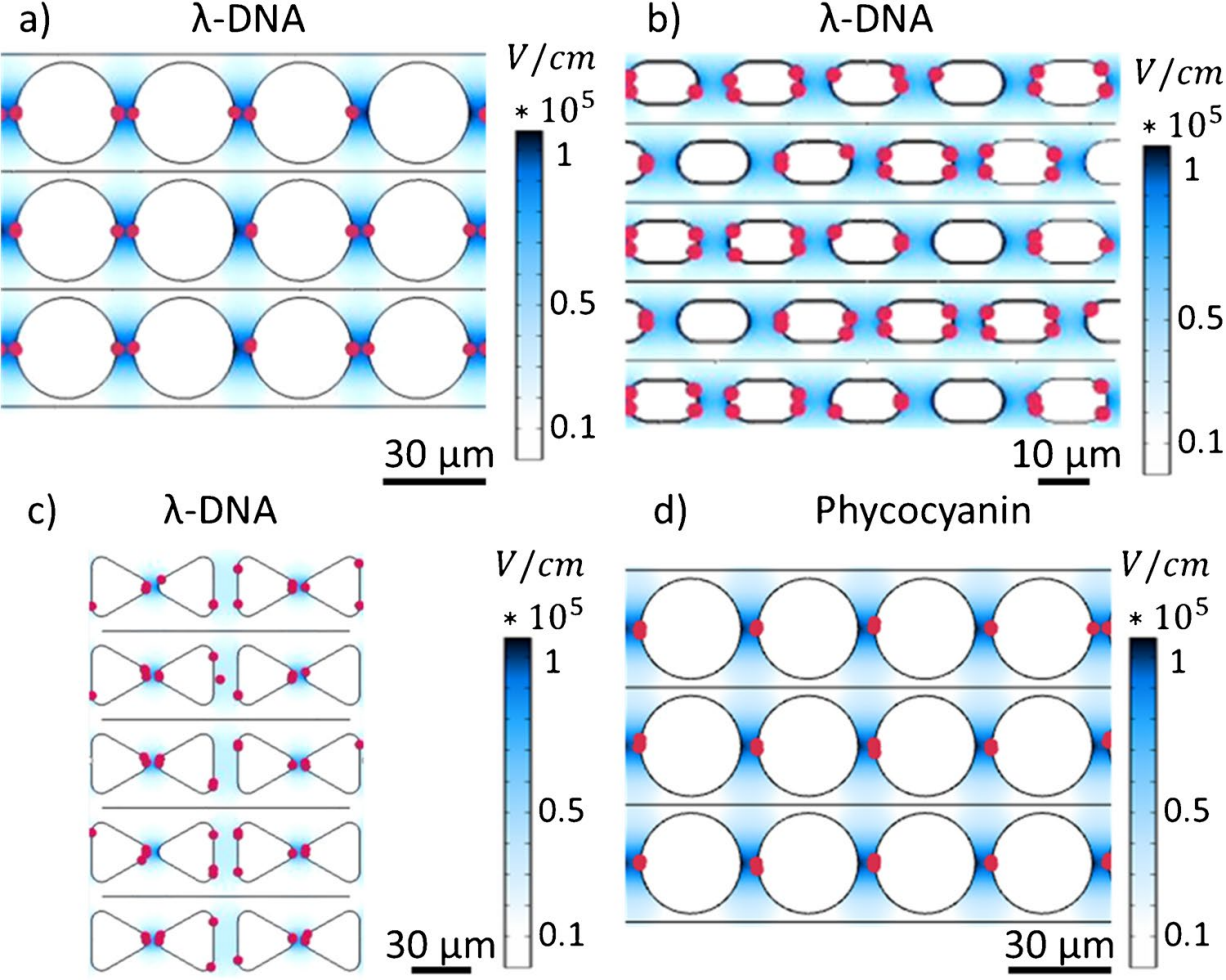
Numerical modeling of DEP behavior for λ-DNA and Phycocyanin. **a**–**c** Result of the particle tracing model for λ-DNA in circular, elliptical, and triangular post geometries at applied potential of 750 V/cm (500 Hz) using $\alpha$ = 3.3*10^–29^ Fm^2^. In the three cases, pDEP trapping was observed. **d** Result of the particle tracing model for Phycocyanin. pDEP trapping resulted with $\alpha$ = 3.5*10^–31^ Fm^2^ at an applied potential of 1000 V/cm (500 Hz). 100 particles were released from the black horizontal lines in each case, and the potential was applied from top to bottom. The horizontal post gap used for circular posts was 5 µm, for elliptical posts was 8 µm, and for triangular posts was 5 µm. The color scheme shows the resulting electric field distribution in the post arrays. For **a**–**d**, the superimposed red dots indicated the final position of the particles released from the horizontal lines

We further studied DEP trapping behavior through a numerical model using particle tracking. This model included the matching post-array geometry with the applied potential scaled to account for the actual geometry of the 3D-printed device sections (see “Materials and methods” section). The DEP force component was integrated using a user-defined force reflecting the analyte polarizability (see Eq. 5). The migration of the analytes was traced in a time-dependent manner after release from horizontal lines between the two post-rows, as shown in Fig. 2. Three different post geometries are displayed for the DEP trapping of λ-DNA, and the circular geometry for Phycocyanin. These geometries were employed as they had been previously used in DEP trapping applications [16, 17, 39, 80, 81] to demonstrate the capabilities of the model.

Figure 2a-c represents the pDEP trapping behavior of λ-DNA in circular, elliptical, and triangular post arrays, respectively. With an application of 750 V/cm at 500 Hz, λ-DNA was observed to be trapped between two posts of the same row, where the strength of the electric field is highest (pDEP) in all three cases. Extracting the $\nabla {{\boldsymbol{E}}}_{\mathrm{max}}^{2}$ from the model resulted in 2.87*10^15^ V^2^/m^3^ for the circular post-array, 6.63*10^15^ V^2^/m^3^ for the elliptical post-array, and 2.55*10^16^ V^2^/m^3^ for the triangular post-array, using an $\alpha$ value of ${3.3*10}^{-29}{\mathrm{Fm}}^{2}$ as found previously for λ-DNA [29]. The corresponding DEP force responsible for DEP trapping resulted in a magnitude of 0.42 to 0.05 pN. These trapping forces can be compared with previous reports on iDEP trapping of DNA. Yokokawa et al*.* reported DEP trapping of λ-DNA (48.5 kbp) using a quadrupole electrode-based microfluidic device [82], where a DEP force of 0.1 pN at a frequency of 1 kHz was obtained. Regtmeier et al*.* also reported pDEP trapping of λ-DNA (48.5 kbp) using an iDEP device with rectangular posts [29]. Their experiments demonstrated a pDEP trapping force of 6.6 fN for λ-DNA over a frequency range of 30–200 Hz. Their result matches the frequency range of 50 Hz–1 kHz, for which a trapping force in the fN range was also observed for ~ 40 kbp DNA, as reported by Chou et al. [83, 84]. The numerical model thus reflected the expected iDEP behavior and is in excellent agreement with previous reports, demonstrating that it is suitable to predict iDEP trapping behavior of DNA.

Using the same approach, we then investigated the DEP trapping behavior of proteins in iDEP post-array geometries. We are motivated by the fact that theoretical models to describe the protein polarizability are only emerging, and experimental evidence describing protein iDEP varies significantly among different studies, leading to inconclusive results on trapping forces [85–87]. Thus, we used the $\nabla {{\boldsymbol{E}}}_{\mathrm{max}}^{2}$ magnitude of 4.55*10^15^ V^2^/m^3^ (corresponding to 1000 V/cm applied along the device) obtained from the model in the circular post geometry to estimate protein polarizability using Eq. 5. We assessed the particle migration for various $\alpha$ values. For a value of ${3.5*10}^{-31}{\mathrm{Fm}}^{2}$, protein trapping was observed, as indicated by the particle end positions resultant from the particle tracking analysis. As shown in Fig. 2d, the particle tracing analysis demonstrated that DEP trapping occurs with this polarizability value corresponding to a DEP force of 0.05 pN. This finding will be discussed in more detail below with respect to the protein trapping experiments in the “Experimental observation of iDEP with proteins in 3D-printed devices” section.

### 3D-iDEP device designs

High-resolution 3D printing enabled by 2PP was used to generate devices with arrays of different post shapes and gaps to generate high electric fields and allow the iDEP manipulation of DNA and proteins. 2PP was explored due to issues with traditional PDMS devices, specifically for high-aspect ratio realizations of post arrays with µm and sub-µm gaps. Figure S2a-b in the Electronic Supplementary Material shows representative SEM images of PDMS post arrays with non-uniform post diameters, with up to a 50% decrease in top diameter compared to bottom post diameter, arising from inconsistent development of the master wafer. Additionally, bending of the posts in the soft lithography or bonding steps contributed to issues, yielding non-functional devices with PDMS. A non-uniformity in the post diameter or bending will give rise to varying electric field gradients around the posts at different channel heights, leading to inconsistencies in the DEP trapping behavior of analytes (see Electronic Supplementary Material Figure S2a-b for an example).

In contrast, 2PP 3D-printed posts were well-defined, had uniform diameters (5% or less size variation), and could be readily printed in higher aspect ratios of > 5:1 (as defined by the height over diameter ratio). Figure S2c-d in the Electronic Supplementary Material shows SEM images of well-defined 3D-printed circular post arrays with 10 µm diameter and 20 µm height. The devices (V2 and V3) used experimentally had a post diameter of 10 µm and a height of 50 µm with the scope of creating much higher aspect ratio structures (up to 100) with minimum shrinkage to variability in dimensions, as shown previously for microtubes and nanopillars using the same 3D printing technique [88, 89].

**Fig. 3:**
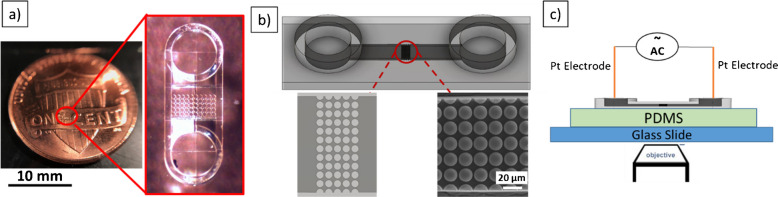
3D-printed iDEP device design and experimental setup: **a** Image of the first 3D iDEP device iteration (V1) with a device length of ⁓1.25 mm. **b** A schematic of the updated 3D-printed iDEP device design (V2) with a total length of ~ 4.5 mm; the left inset shows the post-array design and the right inset image represents an SEM image of the post array showing 2 µm post-gaps. **c** Schematic of the fluorescent imaging setup for the 3D-iDEP experiments. The 3D-printed device was mounted on a thin layer of PDMS slab attached to a glass slide with Pt-electrodes pierced through the reservoirs into the PDMS, as shown in the schematic

Owing to the rapid prototyping capability of 2-PP 3D printing, we have designed several iterations of the overall device and post-array designs and studied these designs using numerical modeling. Figure S2 in the Electronic Supplementary Material shows the major evolution of three different design iterations of the 3D-iDEP device, containing various channel lengths and constrictions to improve the $\nabla {{\boldsymbol{E}}}_{\mathrm{max}}^{2}$. Apart from the effect of the gap distance, as evidenced by Fig. 1, the channel width and shape can improve the electric field acting in the post array. Figure 3a shows an image of the first iteration (V1 in Figure S2 in the Electronic Supplementary Material) device printed with IP-S photoresist, having a total device length of ~ 1.25 mm. This length was chosen for experimental design consideration, allowing for reducing the applied potential but still reaching sufficiently high electric field gradients. The device V1 had an H-gap and a V-gap of 5 µm with a post diameter of 30 µm, but it was too small to be successfully handled during iDEP experimentation since the reservoirs were too close to each other. Thus, the next iteration of the device design (V2 in Figure S2 in the Electronic Supplementary Material) of the 3D-iDEP device with a total length of ~ 4.5 mm is shown in Fig. 3b. The left inset in Fig. 3b shows the post area schematic, and the right inset shows an SEM image of the printed post area. The device was printed with various post gaps, and SEM images revealed that the post areas were well developed with an achievable gap distance down to 2 µm. This emphasizes the rapid and reproducible prototyping abilities of 3D-printed iDEP devices.

For a 5 µm post gap, the device V2 showed a $\nabla {{\boldsymbol{E}}}_{\mathrm{max}}^{2}$ of 2.28*10^16^ V^2^/m^3^, while enclosing the same post array dimensions within a constriction (V3 in Figure S2 in the Electronic Supplementary Material) resulted in the higher $\nabla {{\boldsymbol{E}}}_{\mathrm{max}}^{2}$ of 2.8*10^16^ V^2^/m^3^ [76]. Thus, narrowing or constricting the channel in addition to the post array further increased the acting electric field, which may be advantageous for iDEP experiments, as we will explore below. The small size of the final 3D printed devices required a suitable mount configuration for iDEP experiments. Figure 3c represents the schematic of the experimental setup with the 3D printed device mounted on a thin layer of a PDMS slab attached to a glass slide for fluorescence imaging. The reservoirs were designed to be hollow so Platinum electrodes could be pierced through the underlying PDMS for added stability and connectivity during experimentation.

### Experimental observation of iDEP with proteins in 3D-printed devices

**Fig. 4:**
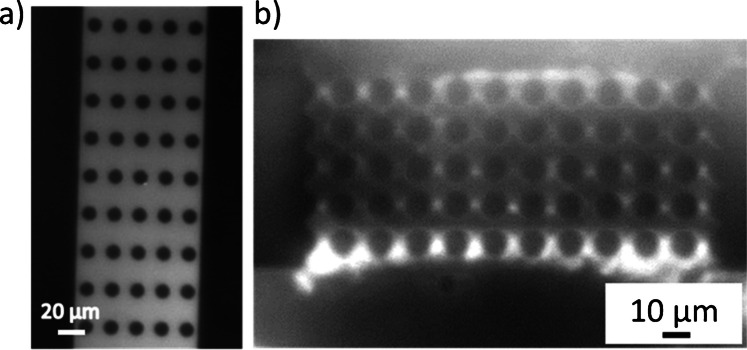
Protein DEP in PDMS vs. 3D-iDEP device. **a** In a PDMS device with 10 µm H-gaps, trapping was not observed for Phycocyanin at 1330 V/cm at 1 kHz; **b** At 960 V/cm and 1 kHz in a 3D-iDEP (V3) device with 5 µm H-gaps, Phycocyanin experiences pDEP as trapping occurs in the high electric field gradient regions indicated by the increased fluorescence intensity in the trapping regions

The high-resolution 3D printed devices were used for the iDEP manipulation of Phycocyanin and contrasted to conventional iDEP devices fabricated via soft lithography in PDMS. Figure 4a, b shows the trapping behavior of Phycocyanin in conventional PDMS-based iDEP devices similar to previous reports [14] and 3D-iDEP devices. Phycocyanin was subjected to 1333 V/cm (at 1 kHz) applied to the PDMS device with H-gaps of 10 µm and V-gaps of 20 µm, corresponding to $\nabla {{\boldsymbol{E}}}_{\mathrm{max}}^{2}$ of 4.05*10^16^ V^2^/m^3^ in the array (Fig. 4a). Trapping-like behavior was not observed, as evidenced by a homogeneous distribution of the fluorescent protein in the entire post array. In contrast, at a lower applied potential of 960 V/cm, pDEP trapping of Phycocyanin resulted in the 3D-iDEP device (V3) due to a higher $\nabla {{\boldsymbol{E}}}_{\mathrm{max}}^{2}$ of 2.57*10^17^ V^2^/m^3^, see Fig. 4b. The higher achieved $\nabla {{\boldsymbol{E}}}_{\mathrm{max}}^{2}$ resulted from a smaller post-gap (5 µm) and the channel constriction (see discussion in the “3D-iDEP device designs” section). The Phycocyanin accumulated in the highest electric field regions, indicative of pDEP. This is evident despite the relatively large fluorescence background arising from the 3D printing photoresin and some crosstalk due to the short device dimension and large sample volume in the reservoir adjacent to the post array.

The applied potential is only 40 V/cm lower compared to the numerical model, indicating good agreement between model and experiment. We further confirmed the presence or absence of trapping by calculating the trapping strength from the background-subtracted fluorescence images. Trapping strength was defined as the ratio of the fluorescence intensity in the trapping regions to that in the non-trapping regions. In the PDMS device, the trapping strength was 0.91, indicating no increase in protein accumulation. In contrast, the 3D-iDEP device exhibited a trapping strength of 5.58, demonstrating a clear accumulation of protein in the pDEP trapping regions. These results experimentally confirm trapping of Phycocyanin in the 3D-iDEP device. We also note that the protein DEP trapping conditions, as represented in Fig. 4b, correspond to the onset of the trapping plateau after reaching the trapping threshold for Phycocyanin, as indicated in Figure S3 in the Electronic Supplementary Material. These observations further indicate that a higher electric field gradient is produced in the 3D-iDEP device due to narrower post gaps and the channel constriction integrated into this device design (V3). We note that the electric fields could be further enhanced with sub-micrometer post-gaps as discussed in the “Electric field gradient as a function of post-gap” and “3D-iDEP Device Designs” sections, such that the required applied potential for DEP trapping can be reduced. Additionally, the IP-S photoresin exhibits high background fluorescence within the studied emission ranges even after deliberate photobleaching of the device (as mentioned in the “3D-printed device fabrication” section). Development of a new 3D printing resin with reduced intrinsic fluorescence is therefore expected to improve the signal-to-noise ratio and will be explored in future work to further optimize the 3D iDEP devices.

The successful demonstration of protein trapping with iDEP in the 3D-iDEP designs, as well as the good agreement with the numerical model, could also be a suitable new method to assess protein DEP behavior in more detail and provide useful quantitative information to refine emerging theoretical models. A useful metric constitutes the comparison of DEP forces required for protein trapping. For example, Hölzel et al*.* reported pDEP trapping of R-Phycoerythrin requiring a trapping force of 0.1 pN. Using the numerical model explained in the “iDEP trapping assessed by numerical modeling” section and the iDEP trapping conditions of Phycocyanin, we estimated that a trapping force of 0.05 pN is required to trap the protein in the 3D-iDEP device. Our estimated DEP force is approximately half of the trapping force estimated by Hölzel et al*.* [69]. Overall, we note that theoretical models for protein DEP are still emerging and that our estimations fall well within the sub-pN range of DEP trapping forces previously reported for proteins [85–87, 90].

### Experimental observation of iDEP with λ-DNA in 3D-printed devices

**Fig. 5:**
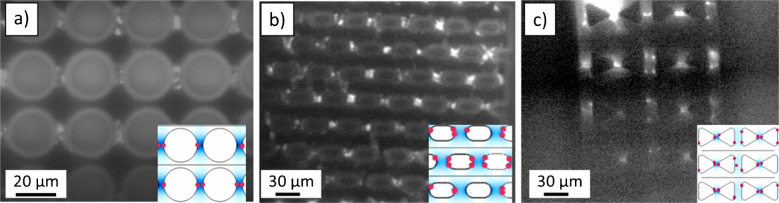
Experimental iDEP trapping in 3D printed devices observed for λ-DNA with numerical model results as insets. **a** Fluorescence imaging of λ-DNA subjected to iDEP trapping, at 700 V/cm (500 Hz), λ-DNA (48.5 kbp) shows pDEP as trapping occurs in the regions of the high electric field gradient in a circular post geometry. **b** Similar to **a** but at 800 V/cm, 500 Hz, λ-DNA (48.5 kbp) shows pDEP as trapping occurs in the regions of the high electric field gradient in an elliptical post geometry. **c** Similar to **a** but at 770 V/cm (200 Hz), λ-DNA (48.5 kbp) shows pDEP as trapping occurs in the regions of the high electric field gradient in a triangular post geometry. Fluorescence intensity of trapped analytes differs due to varying imaging conditions for the three cases

The iDEP trapping of λ-DNA was also studied experimentally using the 3D-iDEP devices. To show the potential of 3D printed iDEP devices, a variety of post array geometries were printed, matching post array geometries as reported previously [16, 17, 81]. First, in the circular post array embedded in a straight channel device (V2) and with a minimum applied potential of 700 V/cm at 500 Hz, λ-DNA was trapped in the regions with higher electric field strength, as shown in Fig. 5a. These trapping regions coincide well with the regions predicted by the numerical model in Fig. 2a (also shown as insets), indicating pDEP. We note that the applied electric field is only 50 V/cm below that used in the numerical model, whereas this small discrepancy can be attributed to buffer additives deviating from the properties of water used in the model. The experimentally applied electric field falls well within the plateau region beyond the trapping threshold, as indicated in Figure S3 in the Electronic Supplementary Material. Overall, good agreement between the model and experiment is observed, confirming that the polarizability value determined in an earlier work by [28, 29] Regtmeier et al*.* is also a good predictor for λ-DNA trapping under experimental conditions used here.

Similarly, an elliptical post array and a triangular post array device were printed successfully using the overall device and channel dimensions of V2. Figure 5b, c shows trapping of λ-DNA in the elliptical post array at 800 V/cm (500 Hz) and a triangular array at an applied potential of 770 V/cm (200 Hz), respectively. In both cases, the experimentally observed pDEP trapping for λ-DNA is in excellent agreement with the numerical models (shown as insets) described in Fig. 2 and previously reported studies [11, 13, 29, 91]. We also assessed the trapping strength for λ-DNA experimentally and compared it to the acting electric field gradients in the three post shapes presented in Fig. 5. Table S3 in the Electronic Supplementary Material shows an overview of the trapping strength with the corresponding $\nabla {{\boldsymbol{E}}}_{\mathrm{max}}^{2}$ computed in COMSOL based on the experimentally applied electric field in each case. The triangular post array resulted in the highest $\nabla {{\boldsymbol{E}}}_{\mathrm{max}}^{2}$ which was an order of magnitude higher than for the other two cases. In accordance, the trapping strength resulted in the highest value (2.54), which agrees with the results from the model (see “iDEP trapping assessed by numerical modeling” section). Experimentally, we found the trapping strength obtained in the circular and elliptical cases to be lower (1.59 and 1.04, respectively), which we attribute to the same order of magnitude electric field gradients acting for the circular and elliptical cases. Future work on improved array geometry, 3D printed with a lower background fluorescence photoresin, should provide further details on optimized geometries in 3D-iDEP devices.

### Sub-micrometer resolution 3D-iDEP devices for nano-scale analytes

The ultimate goal of this study was to develop high-resolution 3D-iDEP devices with nm to µm post-gaps, enabling high electric field gradients required for iDEP-based manipulation of nm-scale bioanalytes, which is challenging and labor-intensive to reproducibly fabricate with PDMS using standard photolithographic approaches. While special formulations of “hard” PDMS have been previously used to create nm-post gaps [42, 59], the hard PDMS formulation is brittle, posing issues during casting steps [59]. 3D printing, however, enables much faster prototyping compared to traditional microfabrication approaches, as it does not require the production of a mask and a resin on a Si master template before imprinting the new design on the fabrication material.

**Fig. 6:**
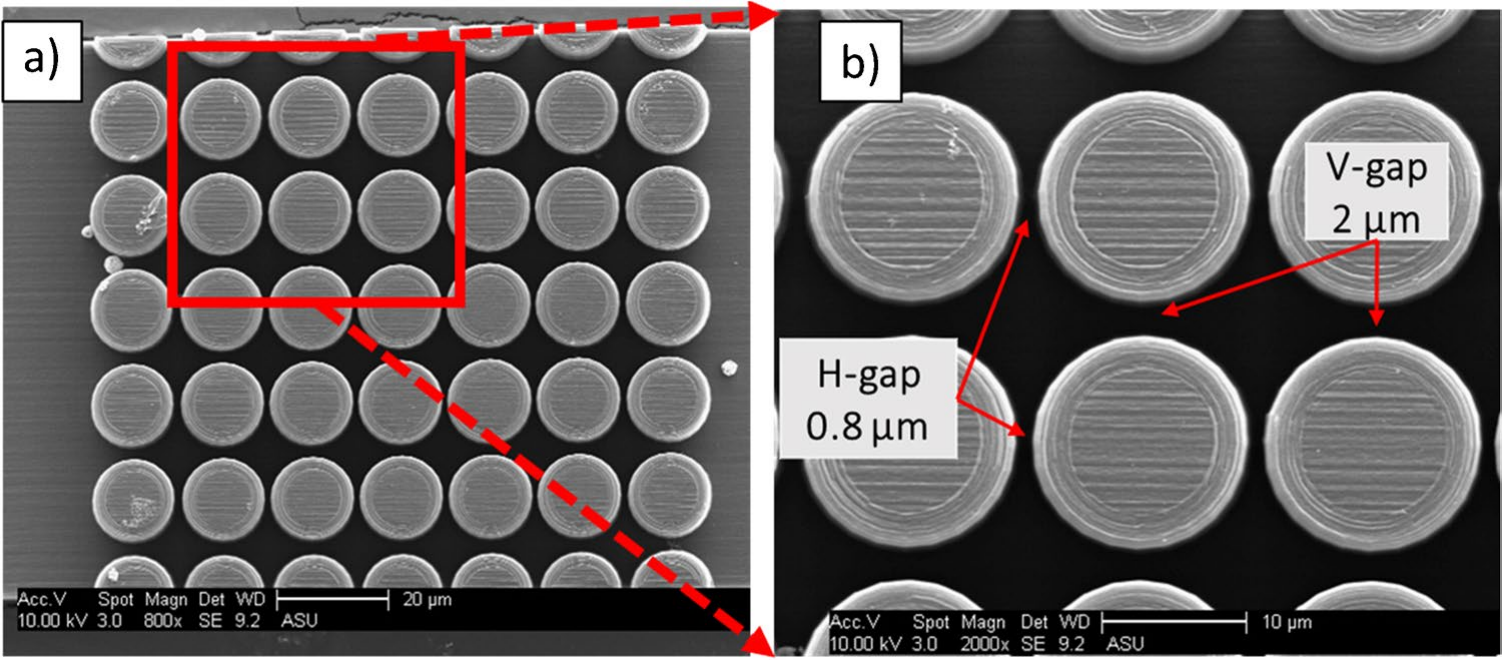
**a** Scanning electron micrographs of a post array with submicron-resolution achieved using IP-Dip photoresist; **b** zoom-in of **a** showing gaps of 2 and 0.8 µm successfully 3D-printed and well-resolved

Thus, to reach even higher electric field gradients over 10^17^ V^2^/m^3^ in post arrays without an additional channel constriction, as shown in Fig. 1, we attempted to decrease the gap distance experimentally. We employed the IP-Dip photoresist and a small-feature printing protocol to achieve improved spatial resolution [62, 92]. Due to the micrometer-sized channels and nano-scale gaps in this design, the development step (removal of unpolymerized photoresist via organic solvents) poses a challenge. Therefore, an open-face post-array device was designed to assess printing parameters and resolution. After optimizing printing parameters such as laser power, scan speed, etc., an open-face post array device with post gaps down to ~ 800 nm was successfully printed and developed as shown in Fig. 6. The high-resolution 3D-iDEP devices can be covered with a PDMS slab to enclose a channel for iDEP applications and are capable of generating $\nabla {{\boldsymbol{E}}}_{\mathrm{max}}^{2}$ in the range of 10^16^ to 10^17^ V^2^/m^3^ with minor design updates, as shown with numerical modeling in Fig. 1. Further design and printing parameter characterization are underway to improve the post-gap resolution and develop a completely enclosed semi-3D-printed device to manipulate nano-scale analytes in the future. A two-step multi-resin 3D-printing approach, where sub-micrometer gap post-arrays are printed and developed first using IP-Dip resin, and the bulk of the microfluidic device is printed around subsequently, may lead to the successful 2PP 3D printing fabrication of 3D-iDEP devices with improved gaps.

## Conclusion

3D printing technologies have been widely accepted and employed in many areas of biological and analytical applications, including microfluidics, due to their rapid prototyping abilities and constantly improving spatial resolution. However, iDEP studies have not been demonstrated in a completely 3D-printed device. We successfully printed and developed 3D-iDEP devices with variable post gaps and diameters, and demonstrated the pDEP trapping for two model biomolecules, the protein Phycocyanin and λ-DNA in a completely 3D-printed microfluidic device. Additionally, numerical models were developed and studied to predict $\nabla {{\boldsymbol{E}}}_{\mathrm{max}}^{2}$ for various post-gaps. The numerical model predicted an over 25-fold increase in $\nabla {{\boldsymbol{E}}}_{\mathrm{max}}^{2}$ when H-gaps were reduced from 10 µm to 100 nm, and an over 125-fold improvement in $\nabla {{\boldsymbol{E}}}_{\mathrm{max}}^{2}$ upon further modification of post-array geometries in terms of PD and V-gaps dimensions. This indicates future potential for improving the maximum achievable electric field gradient by further adapting the post diameters and spacing. Furthermore, the iDEP trapping behavior of Phycocyanin and λ-DNA was also addressed in a numerical model using the polarizability of both analytes. The experimental trapping observations for the two analytes were quantified by measuring the trapping strength, and trapping conditions were found to be in very good agreement with the numerical model. Our work thus also outlines a way of quantitatively obtaining the polarizability of biomolecules through the experimental determination of the trapping condition and subsequent comparison with the numerical model.

3D-iDEP devices with post-gap distances of 800 nm were successfully printed using a specialized IP-Dip photoresin, capable of generating $\nabla {{\boldsymbol{E}}}_{\mathrm{max}}^{2}$ in the range of 10^16^ to 10^17^ V^2^/m^3^. With the continuous advancement in the field of 3D printing, next-generation 2PP printers are being made available commercially that have further improved the resolution and printing speeds by using multi-resin or adaptive mesh technologies [93]. Such advancements in the field of 3D printing further pave the way for nanometer resolution devices with truly three-dimensional geometries, allowing DEP studies of nanoscale analytes, including other proteins, DNAs, and viruses, in the future.

## Supplementary Information

Below is the link to the electronic supplementary material.

Supplementary file1 (PDF 1.89 MB)

## Data Availability

Data presented in the manuscript are available from the authors upon reasonable request.
