# Supplementary material for: High-resolution 3D-printed insulator-based dielectrophoresis devices for biomolecular manipulation
Source: Anal Bioanal Chem. 2026 Jan 25;418(15):4749–62. doi: 10.1007/s00216-026-06331-6 (PMC13388432; doi:10.1007/s00216-026-06331-6)
Supplement: Supplementary file 1 — Supplementary file1 (PDF 1.89 MB) [file 216_2026_6331_MOESM1_ESM.pdf]

Supplementary Material for

High-resolution 3D-printed Insulator-based Dielectrophoresis Devices for Biomolecular Manipulation

[1,2]Mukul Sonker, [1,2]Mohammad Towshif Rabbani, [1,2]Samira Mahmud,[1,2] Jorvani Cruz Villarreal, [1,2]Domin Koh, [2]Raimund Fromme, [1,2]Alexandra Ros*

[1]*School of Molecular Sciences, Arizona State University, Tempe, Arizona, United States*
[2]*Center for Applied Structural Discovery, The Biodesign Institute, Arizona State University, Tempe, Arizona, United States*

Table S1: Parameters used for numerical modeling.

Variable	Value	Unit
Temperature	298	K
Density of water	997	kg/m^3
Dynamic viscosity of water	0.89	cP
Radius of λ-DNA	720	nm
Radius of phycocyanin	3.5	nm
Diffusion coefficient of phycocyanin	$6.9 * 10^{-11}$	m^2/s
Polarizability (α_λDNA)	$3.3 * 10^{-29}$	Fm^2
Polarizability_final (α_phycocyanin)	$3.5 * 10^{-31}$	Fm^2

Table S2: $\nabla \boldsymbol{E}_{max}^2$ and $\boldsymbol{E}_{max}$ for various H-gaps, V-gaps, and Post Diameters at an applied potential of 1000 V/cm in a 140 µm (L) × 80 µm (W) rectangular channel, numerically calculated using COMSOL Multiphysics 6.3.

H-Gap (µm)	V- Gap (µm)	Post Diameter (µm)	$\nabla \boldsymbol{E}_{max}^2$ $(V^2/m^3)*10^{16}$	$\boldsymbol{E}_{max}$ $(V/m)*10^5$
20	10	10	1.2	1.77
15	10	10	1.2	1.79
10	10	10	1.3	1.83
5	10	10	1.6	2.04
4	10	10	1.0	2.17
3	10	10	2.2	2.39
2	10	10	2.9	2.75
1	10	10	5.3	3.74
0.75	10	10	7.1	4.29

0.50	10	10	5.7	5.23
0.25	10	10	20.4	7.18
0.10	5	10	32.1	9.01
0.10	10	10	34.8	9.36
0.10	20	10	94.2	15.3
0.10	10	5	127.0	12.7
0.10	20	5	162.0	15.1

Table S3: λ-DNA Trapping strength comparison for the three post shapes used for the 3D-iDEP devices shown in Figure 5a-c.

Post Shape	Trapping Strength	$\nabla \boldsymbol{E}^2_{max}$ (V^2/m^3)*10^{15}
Circular	1.59	2.5
Elliptical	1.04	7.6
Triangular	2.54	26.6

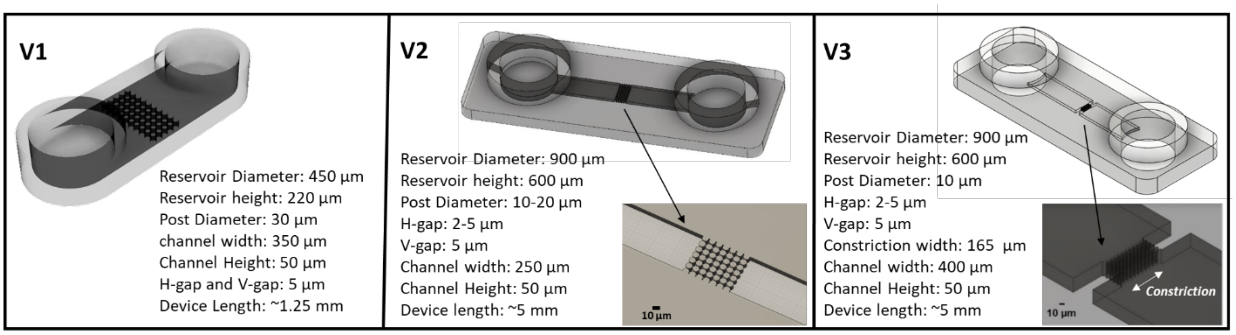

Figure S1: Schematics and dimensions of the major evolution of the overall designs of different 3D-DEP devices (V1-V3) developed during this study.

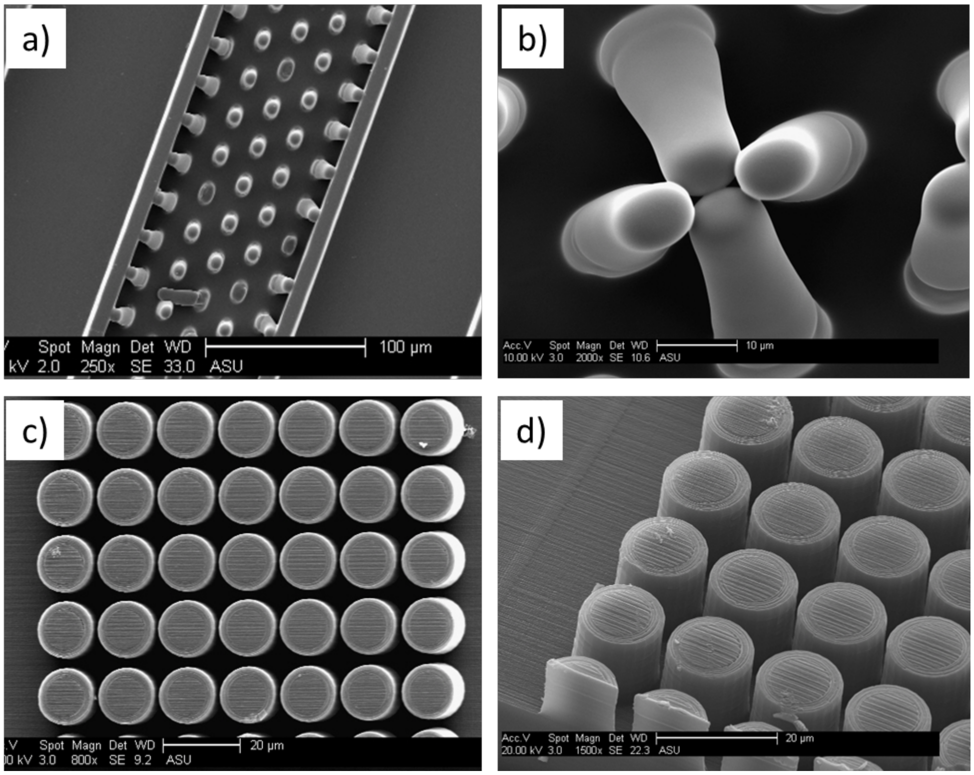

Figure S2: (a-b) Scanning electron micrographs (SEM) of the PDMS devices showing deformities with higher aspect ratio structures. PDMS posts are designed to be elliptical in shape with 17 µm length, 10 µm width, and 20 µm height. (c-d) SEM of a 3D-printed iDEP device showing reproducibly printed post array with 10 µm diameter and 20 µm height with post-gaps of 2 µm.

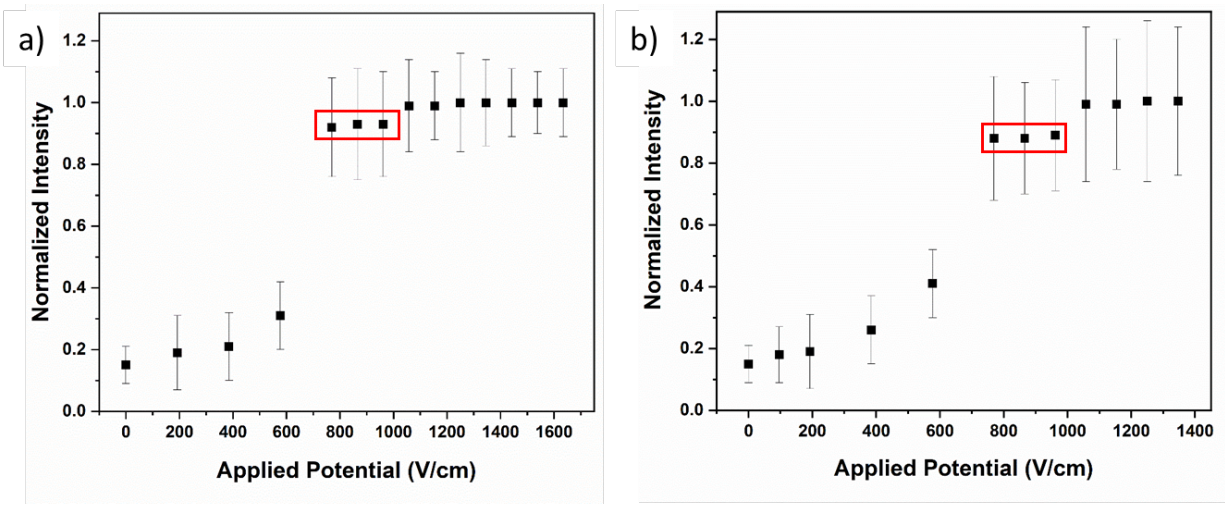

Figure S3: Normalized fluorescence intensity indicative of iDEP trapping as a function of applied electric potential for (a) Phycocyanin (b) λ-DNA, observed in 3D-iDEP device (V3). Red rectangles represent the plateau region, indicating pDEP trapping threshold in the circular post array devices as shown in Figure 4b (Phycocyanin) and Figure 5a (λ-DNA).
